# Update on Biomarkers Associated to Cardioembolic Stroke: A Narrative Review

**DOI:** 10.3390/life11050448

**Published:** 2021-05-17

**Authors:** Ana Catarina Fonseca, Pedro Coelho

**Affiliations:** 1 Department of Neurology, Hospital de Santa Maria, 1640-035 Lisboa, Portugal; peminco@gmail.com; 2 Institute of Molecular Medicine, 1649-028 Lisboa, Portugal; 3 Faculdade de Medicina, Universidade de Lisboa, 1649-028 Lisboa, Portugal

**Keywords:** biomarker, cardioembolism, stroke, atrial fibrillation, patent foramen ovale, atrial cardiomyopathy, NT-proBNP, BNP, ANP

## Abstract

Background: In the last years, several studies were conducted that evaluated biomarkers that could be helpful for cardioembolic stroke diagnosis, prognosis, and the determination of risk of stroke recurrence. Methods: We performed a narrative review of the main studies that evaluated biomarkers related to specific cardioembolic causes: atrial fibrillation, patent foramen ovale, atrial cardiomyopathy, and left ventricular wall motion abnormalities. Results: BNP and NT-proBNP are, among all biomarkers of cardioembolic stroke, the ones that have the highest amount of evidence for their use. NT-proBNP is currently used for the selection of patients that will be included in clinical trials that aim to evaluate the use of anticoagulation in patients suspected of having a cardioembolic stroke and for the selection of patients to undergo cardiac monitoring. NT-proBNP has also been incorporated in tools used to predict the risk of stroke recurrence (ABC-stroke score). Conclusions: NT-proBNP and BNP continue to be the biomarkers most widely studied in the context of cardioembolic stroke. The possibility of using other biomarkers in clinical practice is still distant, mainly because of the low methodological quality of the studies in which they were evaluated. Both internal and external validation studies are rarely performed for most biomarkers.

## 1. Introduction

In many areas of medicine, biomarkers are currently used to help to diagnose, establish prognoses, and to prescribe therapeutics to specific groups of patients that benefit the most from differentiated treatments due to having particular phenotypes [1]. Moreover, in neurology, several studies that evaluated biomarkers have been conducted in recent years, namely in the area of stroke [2]. When compared to other medical areas, the application of biomarkers to the field of cerebrovascular pathology has some obstacles: (a) the presence of the blood–brain barrier that poses difficulties and delays the release of proteins of neuronal or glial origin into the bloodstream after stroke; and (b) many of the potential serum biomarkers of cerebral ischemia and inflammation have low specificity and may also be increased in situations that can be confounded with stroke as they may present in a similar way such as acute myocardial infarction or central nervous system inflammation [3]

Nevertheless, there could be clear benefits of the application of biomarkers in stroke. Unravelling the cause of ischemic stroke can sometimes be challenging [4]. After an extensive investigation about a third of ischemic stroke patients are classified as having an undetermined stroke etiology [4]. It is possible that a fraction of these strokes that are currently being classified as of undetermined etiology may indeed be cardioembolic. Cardioembolic strokes are associated to a high morbidity and mortality [5] and some like the ones associated to atrial fibrillation (AF) and to a patent foramen ovale (PFO) have specific treatments that can reduce the risk of stroke recurrence. 

In this article, we aimed to perform a narrative review of the most recent literature regarding biomarkers associated to cardioembolic stroke. We searched studies that evaluated biomarkers associated to established cardioembolic etiologies like AF and PFO but also associated to emerging new possible causes of cardioembolic stroke, such as atrial cardiomyopathy and left ventricular wall motion abnormalities (LVWMA). We assessed biomarkers associated to diagnosis, prognosis, and stroke risk and recurrence.

## 2. Atrial Fibrillation

Atrial fibrillation is one of the main causes of cardioembolic stroke. Strokes related to AF are associated to a high morbidity and disability [5].

Current guidelines define presence of AF as electrocardiographic documentation of absolutely irregular RR intervals and no discernible, distinct P waves lasting for at least 30 s. However, there is no evidence to suggest that a run of AF lasting > 30 s is more important than several shorter episodes. On the other hand, our understanding of the risk factors and complications of AF is based mostly on studies that have evaluated AF in a binary fashion (present or absent) and have not investigated AF burden (quantity the cumulative amount of AF that an individual has). It is unclear whether the risk increases continuously or whether a threshold exists.

The diagnosis of paroxysmal AF may be challenging. To date, several studies explored the use of prolonged noninvasive and invasive cardiac monitoring devices to identify AF. Nevertheless, the current available methods to detect paroxysmal AF have low sensitivity. It remains elusive which is the best method and the duration of monitoring to detect paroxysmal AF. It is known that a 24-h ECG recording has a higher sensitivity for the diagnosis of paroxysmal AF than a routine 12 lead ECG. In a systematic review [6] the detection rate of paroxysmal AF by a 24–72 h ECG recording was of 4.6%. Due to the low detection rate of this examination, there is some controversy regarding its clinical utility and its routine use in the etiological investigation of ischemic stroke [7]. Serial ECG assessments within the first 72 h of an acute stroke significantly improve detection of AF [8]. Automated analysis of continuous stroke unit ECG monitoring improves paroxysmal AF detection in patients with stroke on stroke units compared with 24-h Holter ECG [9].

Randomized clinical trials like CRISTAL-AF [10] and EMBRACE [11] showed that longer monitoring of the heart rhythm increased the probability of detecting atrial fibrillation in patients initially suspected of having a cryptogenic stroke (AF lasting > 30 s). However, even in the CRISTAL-AF trial in which insertable cardiac monitors (ICM) were used the rate of atrial fibrillation detection did not surpass 12.4% of patients in the ICM group at 12 months [10]. 

A biomarker that could aid in the diagnosis of paroxysmal AF would be very helpful, namely to select patients that could benefit the most from long term heart rhythm monitoring. Several studies have analyzed possible biomarkers of AF. Most have analyzed levels of biomarkers in patients with known AF. Only a few have prospectively analyzed how biomarkers measured at baseline were predictive of AF.

### 2.1. NT-proBNP and BNP

#### 2.1.1. Atrial Fibrillation Diagnosis

The majority of studies regarding possible biomarkers of AF in ischemic stroke refer to the N-terminal of the pro-brain natriuretic peptide (NT-proBNP) and the brain natriuretic peptide (BNP). 

NT-proBNP is part of a group of natriuretic peptides, phylogenetically conserved along time that includes peptides such as atrial natriuretic peptide (ANP), natriuretic peptide type C, urodilatin and the Dendroaspis natriuretic peptide [12]. NT-proBNP is coded by a gene composed by three exons and two introns that is located in chromosome 1p36.2. It is initially produced as a prepropeptide of 143 amino acids. This prepropeptide is proteolytic cleaved into a non-active N-terminal fragment composed by 108 amino acids designated proBNP. ProBNP after secretion is divided in two fractions by two proteolytic enzymes. These two fractions are BNP that is biological active (aa 77–108) and the N-terminal-proBNP (NT-proBNP) (aa 1–76) without biological activity [13]. BNP and NT-proBNP concentrations can be determined in blood samples [14]. The half-life of these two peptides differs. NT-proBNP has a half-life superior to BNP. NT-proBNP has a half-life of 120 min and BNP a half-life of 22 min [15]. It is due to this difference in half-life that most essays measure NT-proBNP instead of BNP. In the heart, BNP can be produced by both atria and ventricles [16]. However, due to the larger ventricular mass, 70% of all BNP is produced by the ventricle in normal conditions [17].

Higher levels of NT-proBNP and BNP have been consistently shown to be present in patients with cardioembolic stroke associated to AF when compared to patients with a noncardioembolic etiology [18,19]. In the first 72 h after ischemic stroke, NT-proBNP serum levels have been shown to have a similar diagnostic accuracy to diagnose cardioembolic stroke, although NT-proBNP levels were highest in the first two days after ischemic stroke and declined significantly thereafter [20].

Several cut-off points for the diagnosis of cardioembolic stroke associated to AF have been proposed. The use of different methodologies and assay kits may hamper the establishment of a cut-off point that could be used in a generalized way. Although NT-proBNP and BNP levels tend to increase with age and differ according to gender, scarce studies searched for different cut-off points according to these characteristics [21]. Moreover, several conditions may increase NT-proBNP and BNP levels and be confounding factors. Therefore, it is important to recognize what pathologies can be associated to increased levels of natriuretic peptides. These include: acute ischemic heart disease, heart failure, heart valve disease, renal failure, anemia, acute pulmonary embolism, pulmonary hypertension, sepsis and hyperthyroidism [22]. Treatment with angiotensin-converting enzyme inhibitors (ACEI), Angiotensin II receptor blockers (ARB), diuretics and Beta-blockers can also increase NT-proBNP and BNP levels [22]. There is an inverse relationship between natriuretic peptide levels and obesity (body mass index ≥ 30) [23].

Only some studies prospectively followed cryptogenic ischemic stroke patients to determine if NT-proBNP levels measured at baseline at the time of ischemic stroke onset could be predictors of a later diagnosis of AF [24]. In one study that analyzed serum levels of NT-proBNP, eighty patients with cryptogenic stroke were followed during six months to look for AF. In 17, paroxysmal AF was found during follow-up. In these patients, the area under the curve for the diagnosis of paroxysmal AF was 0.83. The cut-off point of 265.5 pg/mL had a sensitivity of 88.2% and a specificity of 61.9%. The cutoff point of 912 pg/mL had a sensitivity of 47.1% and a specificity of 88.9% [24].

A systematic review with pooled meta-analysis that analysed NT-proBNP and BNP reported that BNP/NT-proBNP levels were significantly elevated in cardioembolic stroke. Predictive models showed a sensitivity >90% and specificity >80% when BNP/NT-proBNP were added considering the lowest and the highest quartile, respectively. Both peptides also significantly increased the area under the curve and integrated discrimination improvement index compared with clinical models [25].

A clinical trial is currently using NT-proBNP concentrations levels to decide upon further screening for AF [26]. The STROKESTOP II trial is a randomized, population-based study of AF screening in individuals older than 75. All individuals with NT-proBNP > 125 ng/L and without known AF are taught to undergo intermittent ECG recordings twice daily for two weeks (intervention group). Individuals with a NT-proBNP < 125 ng/L do one initial 1-lead ECG, and if normal do not undergo further ECG screening (control group). The primary outcome measure is the incidence of stroke or systemic embolism in the control group versus the intervention group [26].

#### 2.1.2. Stroke Recurrence 

A study that used data from the ENGAGE AF-TIMI 48 which was a randomized trial of the oral factor Xa inhibitor edoxaban in patients with AF and a CHADS2 score of ≥2, evaluated NT-proBNP as a predictor of stroke recurrence in anticoagulated patients [27]. This studied included 6308 patients. In a Cox regression model, upward changes in log2-transformed NT-proBNP were associated with increased risk of stroke or systemic embolic events in anticoagulated patients (adjusted hazard ratio (adj-HR) 1.74; 95% confidence interval (CI) 1.36–2.23 and adj-HR 1.27; 95% CI 1.07–1.50, respectively) [27].

### 2.2. Atrial Natriuretic Peptide (ANP) and Midregional Fragment of the Precursor Hormone of ANP (MR-proANP)

The Atrial natriuretic peptide (ANP) or atrial natriuretic factor (ANF) is a natriuretic peptide hormone secreted from the atria. As it is only produced by the atria in contrast to BNP and NT-proBNP that are both produced by atria and ventricles it could theoretically be a biomarker more accurate to diagnose AF. MR-proANP is a stable fragment of the ANP precursor hormone [28]. A retrospective study compared the diagnostic accuracy of ANP and BNP. Two-hundred and twenty-two consecutive ischemic stroke patients within 48 h after stroke onset were evaluated. Plasma ANP and BNP were simultaneously measured at admission. One hundred and fifty-eight patients had no evidence of AF, 25 patients had paroxysmal AF, and the other 39 patients had chronic AF. ANP was significantly higher in the paroxysmal AF than in the sinus rhythm group (97 (50–157) mg/dL versus 42 (26–72) mg/dL, *p* < 0.05) and further increased in the chronic AF group (228 (120–392), *p* < 0.05 versus paroxysmal AF and sinus rhythm groups). Similarly, the BNP value was higher in the paroxysmal AF than in the sinus rhythm group (116 (70–238) mg/dL versus 34 (14–72) mg/dL, *p* < 0.05) and further increased in the chronic AF group (269 (199–423) *p* < 0.05 versus paroxysmal AF and sinus rhythm groups). The area under the curve was similar between ANP and BNP (0.76 and 0.80) [28].

In a prospective multicenter cohort study that quantified MR-proANP levels in ischemic stroke patients within 24 h of stroke onset, the diagnosis of AF at hospital discharge were associated with MR-proANP with an OR of 18.35 (95% CI 7.94–42.45, *p* < 0.001) [29]. The continuous net reclassification index of MR-proANP for AF was 78%, 95% CI 60%–89%, *p* < 0.001). MR-proANP levels ≥ 289 pmol/L had a specificity of 86% and sensitivity of 48% for the diagnosis of AF.

### 2.3. Interleukin-6

IL-6 is an inflammatory cytokine with pleiotropic effects on immune response and inflammation. It is synthesized in immune cells such as macrophages and monocytes. Emerging studies have proved that inflammation plays a key role in AF. Thrombogenesis related to AF is also associated with inflammation [30].

A meta-analysis of five studies involving 22 928 patients showed that higher levels of IL-6 in AF patients are related to the risk of long-term thromboembolic events including stroke (RR 1.44, CI 95% 1.09–1.90, *p* = 0.01) [30]. A cut-off point of IL-6 needs to be defined.

### 2.4. Growth Differentiation Factor-15, Cystatin and High Sensitive Cardiac Troponin

A new biomarker-based risk score to improve the prognostication of major bleeding in patients with AF was developed by Hijazi et al. [31]. The authors developed and internally validated a new biomarker-based risk score for major bleeding in 14,537 patients with AF randomized to apixaban versus warfarin in the ARISTOTLE trial and externally validated it in 8468 patients with AF randomized to dabigatran versus warfarin in the RE-LY trial. Plasma samples for determination of candidate biomarker concentrations were obtained at randomization. The ABC-bleeding score, using age, history of bleeding, and three biomarkers (hemoglobin, high sensitive cardiac troponin (cTn-hs), and growth differentiation factor-15 (GDF-15) or cystatin C) was internally and externally validated and calibrated in large cohorts of patients with AF receiving anticoagulation therapy. The ABC-bleeding score performed better than the HAS-BLED and ORBIT scores [31]. GDF-15 is a marker of oxidative stress; cardiac troponin is linked to myocardial injury and cystatin C is a marker of renal function. 

An ABC-AF-stroke (age, biomarkers, prior stroke) has also been developed to improve prognostication of stroke in patients with AF. This score incorporates NT-proBNP and cTn-hs levels as biomarkers, age and history of prior stroke/transient ischemic attack. It was developed and internally validated in 14,701 patients with AF treated with oral anticoagulation [32]. Biomarkers levels were evaluated at baseline and patients were followed for a mean time of 1.9 years. An external validation was performed in 1400 patients with AF with a median follow-up of 3.4 years. This score had a better accuracy to predict stroke in patient with AF than the CHADS2DS2VASC score in the derivation and validation cohorts (AUC of 0.68 vs. 0.62 *p* < 0.001 and 0.66 vs. 0.58 *p* < 0.001 respectively) [32].

Some studies have reported higher values at hospital admission of troponins (Cardiac troponin I) in patients with cardioembolic stroke than in patients with non-cardioembolic stroke. However, these studies did not discriminate the subtypes of cardioembolic stroke that were included [33,34].

### 2.5. D-dimers

D-dimers are a breakdown product of fibrin. Baseline elevated d-dimers levels (≥2 μg/mL) were found to be associated to a higher risk of recurrent ischemic stroke in patients with AF with HR of 1.80 95% CI 1.13–2.84, *p* = 0.012 [35]. Some studies have shown that elevated d-dimer levels are an independent predictor of the presence of left atrial thrombus in patients with AF [36,37].

### 2.6. Genetics Biomarkers

RNAs present in the peripheral blood have been studied as potential biomarkers. Changes in RNA expression in the blood of patients with cardioembolic stroke could reflect inflammatory and prothrombotic changes specific to this stroke etiology [38]. Microarray and RNA sequencing or PCR with reverse transcription allow the study of noncoding and coding RNA transcripts. In a discovery-based study that collected blood samples from 76 acute ischemic stroke patients, using whole genome microarrays, a 37-gene profile was able to differentiate cardioembolic stroke due to AF from non-AF causes with > 90% sensitivity and specificity [38]. The identified genes reflected differences in inflammation between stroke subtypes. The major source of RNA in the blood is immune cells including leukocytes, neutrophils, and monocytes. This gene expression signature profile that was identified needs to be validated in future, independent, larger studies [38]. 

MicroRNAs (miRNAs) are single-stranded, non-protein-coding RNAs composed of approximately 22 nucleotides [39]. They are involved in the regulation of post-transcriptional gene expression by binding with the target mRNA at the 3′ untranslated region. Changes in miRNA expression level are reported to be associated to different pathologies (39). The modulation of miRNA expression has been reported to reduce or increase the susceptibility to develop AF in vivo. This may be due to the regulation of atrial remodeling mechanisms by miRNAs [39]. miRNAs could therefore not only be a biomarker but also a potential therapeutic agent for the treatment of AF [40]. 

#### Treatment Implications

Biomarkers associated to the diagnosis of AF could be useful to select patients that could benefit the most from long term heart monitoring. The ideal way to test this possible application would be through a clinical trial, as is currently being tested in STROKESTOP II [26].

The accuracy of established risk scores for stroke, such as the CHA2-DS2-VASc score and the HAS-BLED could potentially be further improved by the addition of serum/plasma biomarkers.

## 3. Patent Foramen Ovale

The foramen ovale is a flap-like communication between the right and the left atria at the level of the fossa ovalis, which is present in the fetus and enables the venous blood to bypass the non-functioning fetal lung and go directly to the left side of the heart [41]. The communication usually closes after birth however if the foramen persists after the age of one, it is called a patent foramen ovale (PFO). It can be found in up to 27% of the general population [41]. Case–control studies demonstrated an association between PFO and cryptogenic stroke (OR 2.9) especially in young adults (<55 years of age) [41,42]. In the last years, results of clinical trials reported a consistently higher benefit of PFO closure compared to antiplatelet therapy in patients with an atrial septal aneurysm or large right-to-left shunts [41]. Some studies were conducted to analyze how biomarkers may help in the diagnosis of PFO or to predict the size of the right-to-left shunt or ischemic stroke recurrence.

### 3.1. L-Arginine/Asymmetric Dimethylarginine Ratio

Sgarra et al. evaluated the correlation, in terms of severity, between the degree of endothelial dysfunction assessed by the L-Arginine/asymmetric dimethylarginine ratio (L-Arg/ADMA ratio), the methylene tetrahydrofolate reductase (MTHFR) genotype, and the interatrial septum (IAS) phenotype in subject with a history of stroke [43]. L-arginine is the substrate for NO generation and asymmetric dimethylarginine (ADMA), an endogenous inhibitor of nitric oxide (NO) synthase. The study included 57 patients, and 10 had a septum integrum (SI), 38 a PFO, and nine an ostium secundum (OS). In the PFO subgroup a negative correlation was found between the L-Arg/ADMA ratio and PFO tunnel length/height ratio (*p* ≤ 0.05; r = −0.37; R^2^ = 0.14). The authors suggest that the L-Arg/ADMA ratio could be used as a reliable marker of stroke susceptibility in carriers of an interatrial septum abnormalities, hinting at its potential use both as a diagnostic tool and as a decision aid for therapy [43]. 

### 3.2. Homocysteine

The 10-point risk of paradoxical embolism (RoPE) score is an index that was created to stratify cryptogenic stroke patients with PFO by their likelihood that the stroke was related to their PFO [44]. Zuin et al. found a direct correlation between serum homocysteine levels and the RopE score in a sample of 244 PFO patients [45]. A receiver operating curve identified the optimal cutoff value of 19.5 μg/dL for homocysteinemia as a predictor of a high RoPE score > 7 in the group of patients with PFO that underwent a closure procedure (AUC 0.90, 95% CI 0.81–0.94, *p* < 0.0001). Multivariate logistic regression analysis demonstrated that a homocysteine serum level ≥ 19.5 μg/dL predicted a RoPE score > 7 (OR 3.21, 95% CI 2.82–3.26, *p* < 0.0001) in closed patients independently from the presence of a permanent right-to-left shunt (OR 2.28, 95% CI 2.01–2.43, *p* = 0.001) and atrial septal aneurysm (OR 3.04, 95% CI 2.64–3.51, *p* < 0.0001) [45].

### 3.3. D-dimers

Kim et al. [46] followed 241 patients with PFO for a mean time of 34.0 ± 22.8 months. During follow-up, all-cause death occurred in 58 (10.2%) patients, ischaemic stroke in 33 (5.8%), and pulmonary thromboembolism in 6 (1.1%). Multivariate Cox regression analysis showed that a D-dimer level of > 1,000 ng/mL was an independent predictor for recurrent ischaemic stroke in patients with PFO (hazard ratio 5.341, 95 % CI1.648–17.309, *p* = 0.005), but not in those without a PFO.

#### Treatment Implications

None of the biomarkers that was reported in the studies analyzed was further validated. Therefore, none can be used in clinical practice.

## 4. Atrial Cardiomyopathy

### 4.1. Introduction

Atrial cardiomyopathy, as defined by a consensus expert group in 2016, consists of any complex of structural, architectural, contractile or electrophysiological changes affecting the atria with the potential to produce clinically-relevant manifestations [47]. This new definition was a landmark and expanded the concept of cardioembolism, including primary atrial disorders as well as providing a framework for integrating the effects of diverse upstream cardiac and systemic disorders that induce secondary atrial remodeling [48].

The actual prevalence of atrial cardiomyopathy is unknown but the extensive list of associated predisposing factors, such as aging, high blood pressure, obesity, diabetes mellitus, and obstructive sleep apnea, suggest that this is a frequently encountered and important clinical condition [47,48]. Furthermore, there is growing evidence that supports the role of atrial cardiomyopathy as an independent determinant of stroke risk, particularly of embolic stroke subtypes [47,49,50,51].Several biomarkers of left atrial dysfunction have been proposed for the diagnosis of atrial cardiopathy, with most of the literature referring to left atrial size (left atrial enlargement), P wave dispersion, P-wave terminal force in lead V1 on EKG, paroxysmal supraventricular tachycardia, atrial high rate episodes, atrial fibrosis, and elevation of serum biomarkers associated with atrial dysfunction like NT-proBNP [52,53,54]. However, it should be noted that some of these biomarkers are not specific to atrial dysfunction [52].

### 4.2. NT-proBNP

N-Terminal pro-Brain Natriuretic Peptide (NT-proBNP) has been shown to be associated with ischemic stroke, particularly cardioembolic stroke, independently of AF [55,56,57]

In a study that used the SPOTRIAS dataset, among 40 patients with cryptogenic stroke, 63% had atrial cardiopathy as evidenced by the presence of at least one of the biomarkers previously described. The most common finding was an elevation of NT-proBNP > 250 pg/mL, present in 49% of cryptogenic stroke patients [58].

Although there is concurrent evidence that a NT-proBNP elevation is associated to an increased likelihood of detecting paroxysmal AF in patients with ischemic stroke [59], bearing in mind that with prolonged heart rhythm monitoring AF detection increases up to 15–30% in cryptogenic stroke [10,11], that could imply that 19–34% of patients may have an atrial cardiopathy without AF. The proportion of NT-proBNP elevation is probably not solely explained by the effect that AF can have on NT-proBNP elevation, highlighting the potential of NT-proBNP in atrial cardiopathy and cryptogenic stroke.

### 4.3. Genetics Biomarkers/Genetic Diseases Associated to Atrial Cardiomyopathy

Variants in genes that are functionally active in the cardiac atria and that contribute to atrial development and/or maintenance of atrial electrical, structural, and metabolic properties may directly cause or modify susceptibility to atrial cardiomyopathy [48].

However, the role of genetics in atrial cardiomyopathy is still in its infancy, and is mostly useful in families with Mendelian forms of disease. For most people, the role of genetics for the prediction of individualized risk of atrial cardiomyopathy and adverse outcomes such as AF has yet to be established [48].

#### 4.3.1. Natriuretic Peptide Precursor A Gene

The natriuretic Peptide Precursor A (NPPA) gene is responsible for the expression of the atrial natriuretic peptide (ANP), an important molecule involved in atrial electric remodelling prevention; intravascular blood volume and vascular tone regulation and ion channel function regulation [60]. Fluctuations in ANP expression due to gene mutations can disturb these mechanisms, contributing to atrial dysfunction. Indeed, atrial dilated cardiomyopathy has been identified in patients with homozygous mutation in NPPA, presenting with severely decreased levels of ANP and with clinical onset in adulthood of biatrial dilatation, early supraventricular arrhythmias with progressive loss of atrial electric activity to atrial standstill and secondary thromboembolic complications, while having a stable normal left ventricular function and long-term stable functional class [46,61]. Patients with this condition require pacemaker or cardioverter defibrillator implantation and chronic anticoagulation because of the high prevalence of thromboembolic complications, most of them consisting of cerebral embolic episodes [61].

#### 4.3.2. Myosin Light-Chain 4 Gene

Myosin light-chain 4 gene (MYL4) encodes the atrial-selective essential myosin light chain. This essential myosin light chain is also known as atrial light chain 1, and is expressed in cardiac and skeletal muscle of the fetus and in the atria after birth, being a key sarcomeric component and important atrial specific contractile protein [62,63]. Mutations in MYL4 gene have been implicated in a primary atrial cardiomyopathy characterized by atrial selective abnormalities associated with conduction system disease, stroke, and eventually mild ventricular dysfunction [62,63]. At least three kindreds have been described: an Icelandic kindred presenting with atrial cardiomyopathy, with a frameshift mutation in *MYL4* associated with early-onset familial AF and bradyarrhythmias requiring pacemaker insertion, ischaemic stroke and sudden death [62], a Canadian kindred presenting with early-onset AF, conduction disease and reduced left atrial function as measured by left atrial function index [64], and a Chinese kindred presenting with an inherited atrial cardiomyopathy and atrial standstill as a prominent feature, evolving from contractile abnormalities at a young age, followed by atrial arrhythmia, atrioventricular block, and finally total atrial standstill [65].

##### Therapeutical Implications of Atrial Cardiomyopathy

Atrial cardiopathy can be an important cause of cryptogenic stroke and this emerging concept can have important therapeutical implications [66]. The evaluation of atrial cardiopathy can identify patients with a thromboembolic prone left atrium, which could lead to a cardioembolic stroke in the absence of AF [50,66,67]. Taking this into account, anticoagulant therapies may well be of benefit for stroke prevention in a wider population of patients than just those with AF. Preliminary evidence of this effect can be seen in an analysis of the WARSS trial, in which a subgroup of patients with elevated NT-proBNP had a substantially lower risk of recurrent stroke or death when treated with warfarin rather than aspirin [57]. This result will be further elucidated trials such as the ARCADIA and ATTICUS trial, which could prove the potential role of oral anticoagulation (apixaban versus aspirin) for patients with cryptogenic stroke and atrial cardiopathy [66,67]. To be included in the ARCADIA trial patients have to fulfill at least one of three criteria which include a Serum NT-proBNP > 250 pg/mL (NT-proBNP criterion), P-wave terminal force > 5000 uv x ms in ECG lead V1, and left atrial diameter index ≥ 3 cm/m^2^. The ATTICUS trial that will compare apixaban versus aspirin to prevent ischemic stroke recurrence does not used serum biomarkers but electrocardiographic and echocardiogram parameters suggestive of atrial cardiomyopathy to include patients namely one of the following: LA size > 45 mm (parasternal axis), spontaneous echo contrast in the left atrial appendage (LAA), LAA flow velocity ≤ 0.2 m/s, atrial high rate episodes, CHADSDS2-vass score ≥ 4, FPO [67].

## 5. Left Ventricular Wall Motion Abnormalities

Left ventricular wall motion abnormalities (LVWMA) are a common finding detected on echocardiographic evaluation of stroke patients. These changes are related with various pathologies, such as coronary artery disease, congestive heart failure, stress-induced cardiomyopathy, myocarditis or chronic renal disease [68]. It is believed that left ventricular akinetic or hypokinetic segments could lead to a vulnerable environment where thromboembolism may occur, induced by regional blood stasis and incomplete ventricular emptying [66,69]. However, LVWMA role in stroke is still uncertain [67].

### 5.1. NT-proBNP

There is evidence relating NT-proBNP elevations and cardioembolic stroke [15,16]. However, its applicability for determining the presence of LVWMA is less well established. Hosomi et al. studied the levels of BNP and particular left ventricle segment asynergy in patients who had an ischaemic stroke and had a previous history of an old myocardial infarction [70]. Patients with AF were excluded, and based on their results, cardioembolic stroke was associated with a high plasma BNP level, independently of a left ventricular ejection fraction less than 40% [70]. High plasma BNP levels (> 206.9 pg/mL) and left ventricular wall motion abnormalities in the segments perfused by the left anterior descending coronary artery or right coronary artery showed a high risk for cardioembolic stroke in patients with old myocardial infarction. 

### 5.2. Genetics

LVWMA can be found in patients with cardiac pathology, namely in familial cardiomyopathies such as hypertrophic cardiomyopathy and dilated cardiomyopathy [71,72]. Hypertrophic cardiomyopathy (HCM) is a genetically determined heart muscle disease that is inherited in an autosomal dominant pattern in more than 90% of cases [73]. Over 1500 mutations in at least 15 sarcomere-encoding genes have been identified [74]. About 70% of these mutations are in the sarcomere genes encoding cardiac β-myosin heavy chain (MYH7) and cardiac myosin binding protein C (MYBPC3) [73]. Mutations in troponin T (TNNT2), troponin I (TNNI3), and α-tropomyosin (TPM1) are relatively uncommon causes of HCM and together are responsible for less than 10% of cases [75,76]. Mutations in cardiac α-actin A(CTC1), myosin light chain 2 (MYL2), myosin light chain 3 (MYL3), and Cysteine and Glycine Rich Protein 3 (CSRP3) are other known rare causes of HCM [75].

Dilated cardiomyopathy (DCM) is a heart-muscle disorder characterized by systolic dysfunction and dilatation of the left ventricle with normal left ventricular wall thickness [77]. Familial genetic forms of DCM represent 20-40% of patients [77,78]. Up to 40 genes have been implied in DCM, inherited primarily in an autosomal dominant pattern [77,79]. These genes encode for a wide variety of proteins of the sarcomere, cytoskeleton, nuclear envelope, sarcolemma, ion channels and intercellular junctions [77]. The most common mutations involve sarcomere proteins, in particular titin (TTN) truncating mutations, which are responsible for *25*% of familial DCM [77]. Other sarcomere genes mutated in DCM are MYH7 (encoding myosin 7, also known as β-myosin heavy chain), MYH6 (encoding myosin 6, also known as α-myosin heavy chain), MYBPC3 (encoding cardiac-type myosin-binding protein C), TNNT2, TNNI3, TPM1, MYL2, MYL3 and ACTC1. The most common gene mutated involving nuclear envelope is LMNA, encoding the intermediate filament proteins lamins A and C and responsible for 8% of familial DCM [77,78,79].

#### Therapeutical Implications of Left Ventricular Wall Motion Abnormalities

Left ventricular wall motion abnormalities are an important finding in echocardiographic study of ischaemic stroke patients and may be an independent predictor of stroke recurrence [68,69]. Moreover, LVWMA can present as an echocardiographic finding due to previous cardiac disease, namely myocardial infarction. Anterior apical MI is more likely to result in LV thrombus formation and is associated with cerebral embolism [80]. The detailed role of LVWMA could be assessed in trials evaluating direct oral anticoagulants for prevention of stroke recurrence or systemic embolism in patients with embolic stroke of undetermined source, especially in the analysis of subpopulations with LVWMA.

## 6. Conclusions

Several biomarkers related to cardioembolism are currently being studied (Table 1).

However, NT-proBNP and BNP are still the biomarkers that have been most studied in the context of cardioembolic stroke and for which more evidence exists. Various studies and meta-analysis evaluated these substances as biomarkers of cardioembolic stroke or as predictors of AF. More recently, NT-proBNP and BNP have been used in clinical trials for the selection of patients. These peptides also are starting to be incorporated in risk prediction scores. The majorities of other substances that are currently being evaluated as biomarkers related to cardioembolism, however, are far from being able to be used in clinical trials. This is mainly due to the low methodological quality of most of these studies. Frequently, only a small number of patients are included (<100 patients), and the number of studies that have either an internal or external validation of the cut-off points is very low. Greater adherence to methodological criteria could help to translate results to clinical practice.

## Figures and Tables

**Table 1:** Summary of biomarkers related to cardioembolic sources and the application in which they are currently being evaluated.

Cardioembolic Source	Biomarker	Studied Application
Atrial fibrillation	NT-proBNP/ BNP	Diagnosis
		Stroke recurrence
	ANP/MR-proANP	Diagnosis
	GDF-15	Major bleeding
	cTn-hs	Stroke risk Major bleeding
	IL-6	Stroke risk
	D-dimers	Stroke recurrence
	RNA	Diagnosis
Patent foramen ovale	L-Arginine/asymmetricdimethylarginine ratio	Stroke risk
	Homocysteine	Stroke risk
	D-dimers	Stroke recurrence
Atrial cardiomyopathy	NT-proBNP	Diagnosis
	NNPA	Diagnosis
	MYL4	Diagnosis
Left ventricular wall motion abnormalities	NT-proBNP	Stroke risk
	Genetic markers	Diagnosis
